# The Duty of Medicine to the Race

**Published:** 1930

**Authors:** C. Ferrier Walters

**Affiliations:** Senior Surgeon, Royal Infirmary, Bristol


### The Bristol Medico-Chirurgical Journal

*" Scire est nescire, nisi id me
Scire alius sciret."*

SPRING, 1930.

### THE DUTY OF MEDICINE TO THE RACE.

The Presidential Address, delivered on 9th October, 1929, at the opening of the Fifty-seventh Session of the Bristol Medico-Chirurgical Society.

BY

C. FERRIER WALTERS, F.R.C.S.,

*Senior Surgeon, Royal Infirmary, Bristol.*

I MUST first express my great appreciation of the honour the Society has done me in exalting me to this Chair, inadequately as I can but hope to fill it, in view of those illustrious ones who have preceded me.

As the subject of my address I propose to consider the question of " The Duty of Medicine to the Race."

Man, we have been long assured, has arisen from a simian ancestor, though of late the *tarsius*, that little exophthalmotic creature, has been invoked as his forerunner. If there be any who doubt this they can but admit that *Pithecanthropus erectus*, Piltdown, Rhodesian, Heidelberg and Neanderthal man, unlovely objects as they are depicted, were certainly of his forebears ; while in Cromagnon man of 25,000 years ago—and who knows how much more ?—lived a race they might well be proud of as ancestors. He was

B

not only the equal of present-day man, but certainly surpassed him physically and, if cranial content counts for anything, was his equal if not his superior mentally, certainly his equal in artistic ability, and but that he lacked the accumulated powers, the result of scientific advance, might have outstripped him at the present day.

Whether we assume the Cromagnon as the source of civilization, spreading from the not too fabulous island of Atlantis to Egypt and South America, or prefer the sudden predynastic appearance of civilization in Egypt from an unknown source—though according to Woolley it was from the Indus through the Sumerians that it arose—is of little import compared with the development of civilization, no matter whence it came. Suffice that through the community, the tribe, the city and the state came the great civilizations, some eight or nine in all that we know, rising and falling through a definite period of years, culminating in our own western civilization, foredoomed also to fall, unless we learn from the past and correctly apply our modern scientific knowledge to prevent its decline.

Those past "cycles of civilization" — those "parabolas of peoples"—were ephemeral things, here to-day and gone to-morrow. The 1,800 years of their cycle is as a moment. Gone are the civilizations of Sumer, Egypt, Greece, Crete, Rome, Arabia and Spain. Zimmern likens modern man to the Greeks in mental attainments, as the negro to ourselves at the present day. Is man, therefore, likely to make western civilization any more permanent than those past until and unless he be master of his fate ? We have some evidence of the cause of this decline and fall of races. Certainly in those past perpetual warfare must have been a factor, with its destruction of the fit and survival of the unfit ; a reversal of natural selection.

Greece fell with the decay of marriage and family life, with the psychological infertility of its best stock, with exile and colonization, both in relatively large numbers, with the encouragement of pederasty, above all with the almost perpetual warfare of Greek against Greek, and finally their exportation by their Roman conquerors. All these things tended to eliminate the good stock. Was it a matter for surprise that the collapse of Greece was so sudden and complete?

Despite the fact that Rome drew fresh blood from many sources, a similar degeneration of the people occurred, because of the decline of marriage, the burden that children became and the infertility of the more intelligent classes. Constant warfare was a factor, and the encouragement given to the so-called "proletariat" or producers of offspring. These were the vagabonds, paupers and degenerates, who were encouraged to propagate, for they were exempt from all civil or military duties and from taxation. Rome paid the penalty.

The decline of Arabian civilization is a good example of the effect of perpetual warfare and of miscegenation of an able people. Marriage was allowed with any race, black or otherwise, provided the Mahometan faith was embraced.

With the birth of each civilization was sown the seed of its dissolution. Need this always be? With our present scientific knowledge can this decline not be stayed or even reversed? The inevitable source of the dissolution of past civilizations has been the degeneration of its human material, which has been supplanted by an alien and more virile stock. Until man shall root out and prevent the bad, shall encourage and cultivate the good human element, so long will civilizations be and cease to be. Dare we assume that there will be no cycle to western civilization?

Hope for our civilization dawned with the scientific era, but unfortunately with the overwhelmingly rapid growth of knowledge and the accumulation of scientific facts arises the possibility of a still more rapid decline of man, both mentally and physically, by their unscientific application. Our knowledge has outrun our wisdom. The making and the marring of the race lies in the hands of science. So far, from civilization's point of view, the scales appear to be weighted heavily on the marring side. Even the many physical wonders of to-day, the many apparent advances in medicine, may be powerless to delay for one moment the inevitable. They may serve only to precipitate that descent into oblivion which has overwhelmed past civilizations, unless the application of scientific discovery ceases to be uncontrolled. The necessary conditions for the rise and maintenance of a race are well known—a virile stock, exhibiting good physical and high intellectual qualities, conjoined with initiative, aided perhaps by environment, *but based and grounded on heredity.*

What is this grounding of a successful race on good heredity ? Heredity is described as the mental and physical characteristics transmitted by parents to their offspring. What proof have we that such transmission exists, and that the good and bad within us is handed on to the next and future generations ? A complete handing on of all characteristics does not occur. If it did we might label it with the symbol 1·0, whereas hereditary transmission does not exceed 0·5, which is called the "coefficient of heredity."

Does a race advance or decline by virtue of heredity or environment, by nature or by nurture ? Does what is bred in the bone come out in the blood ? Is a man a chip of the old block ?

As Johnson says: "The social workers of to-day imagine they are improving the race by improving the conditions under which the people live. Few things are farther from the truth. Human nurture as opposed to Nature—in other words, cultural environment—has very little permanent influence on human stock. All experiment and observation offer evidence that the characteristics which an individual inherits from his parents remain remarkably constant under all ordinary conditions to which they are submitted. That constancy appears the more marked the higher we ascend in the scale of evolution. Low forms of life may be made to show change by outside influence. In the higher and more differentiated forms, however, it is found more and more difficult to make any change in them. Their characteristics are more firmly fixed at birth. When we arrive at the highest form, man, even such powerful influences as education and nutrition, varied within their ordinary limits, have *no* power to alter his inborn capacities due to heredity. A man may add by nurture a certain amount to his muscles, but there is an hereditary limit beyond which he cannot develop them. The same holds in games of skill. A certain proficiency may be reached, but the great exponents are born. Environment merely brings them to full fruition and increases their lead over those less fortunately bred."

A dozen men may be crammed to the same extent for an examination. Will their marks be the same? Nature enables the more capable to outstrip the less still further.

Galton, in his study of ordinary and identical twins, found that the former differ widely in capacity and temperament, despite being under the same environment; whereas the identical twins, however different their nurture, remain so alike that their own mothers cannot tell them apart. They exist with but one thought, word and deed between them. If nurture held as the prime factor in development the opposite should hold good. Nurture fails entirely to alter the inborn nature which prevails.

An interesting example of the persistence of inborn good and evil is seen, among many of its kind, in Dugdale's history of the Kallikak (or good-bad family). Martin (Kallikak), a young soldier of good stock, had an illegitimate son by a feeble-minded servant girl. Several years later he married a woman of good family, by whom he had several children. The legitimate children all turned out well and founded one of the most distinguished families in New Jersey. In this family and its branches is found every good type of citizen—judges, lawyers, doctors, educators, traders and landholders. There were no immoral women and only one man sexually loose. In contrast to this, from the illegitimate son of the feeble-minded girl 480 descendants have been traced: 143 were obviously feeble-minded, 33 were prostitutes, 24 confirmed alcoholics, 36 illegitimates, 3 epileptics, 82 died in infancy, there were 3 criminals and 8 brothel keepers. Is this contrast the result of nature or of nurture? Would environment have changed the superior people to inferior and vice versa? The slum man remains the slum man, and will convert any environment into a slum; he is born, not made.

Popenoe and Johnson, in *Applied Eugenics*, say: "There is no evidence worthy of serious consideration that proves the transmission of an acquired bodily characteristic. Lamarck has few supporters nowadays. Nearly all evidence is adduced from mutilations, diseases, the results of use or disuse, and physicochemical effects or environment. Women are not born with pierced ears or Jews without foreskins; the dog and sheep, despite generations of docking, still arrive in this world with the usual appendage; the feet of Chinese children show no deformity at birth. There is ample explanation of evolution in the abundant supply of germinal variations which every individual presents. The same holds good of purely mental processes, such as instincts. There is no proof that habit often repeated becomes instinct, and is handed on by the individual to his descendants, to become racial instinct. The inheritance of acquired characters need not be reckoned with in applied Eugenics."

As Poulton says: "The achievements of the race, and its accumulated social experience, which should be called tradition, may be handed on, but must not be confused with heredity. Whatever wisdom, natural gain or language is passed on from one generation to the next—one generation stands on the shoulders of the other."

But for biological heredity each generation must always start from scratch. Biological improvements can only be made through selective mating.

Jean Jacques Rousseau popularized the idea that all men are born equal, and the Socialists still cling to it. There is no greater fallacy. Nothing is more obvious, on the slightest investigation, than that every grade of mental and physical ability exists, from the genius to the idiot, the athlete to the paralysed; and yet the environmentalists maintain that nurture will efface these differences. Equal opportunity is useless, it serves but to increase the lead of inborn ability.

The fundamental physical and mental differences in each and every human being are inborn. As Stoddart says: "There is an iron law of inequality in man.

It is due to heredity, with the fortuitous variations that accompany it throughout the organic world."

Galton in 1869, in his *Hereditary Genius,* first investigated the production of superior people. From a careful statistical investigation of a great number of famous Englishmen, Galton found that an eminent father was infinitely more likely to have a distinguished son than a mediocre parent. Of many cases cited he found that the son of an eminent judge had a one in four chance of being distinguished, whereas for the son of a man picked haphazard from the population the chance was only about one in four thousand. That this is not environmental, and due to the fact that the obscure man never gets a chance, Galton showed from the history of the papacy. Where it was the custom of the Pope for centuries to adopt a nephew as son and advance him in every way, if opportunity is all that is necessary these men should have reached distinction in the same proportion as the sons of eminent men; but they reached eminence only such as would be reached by the nephew of a distinguished man, which is much less than that of a son. Galton found that half the great men of England had a distinguished close relative.

Wood's statistics in America, where caste lines are not supposed to exist, confirmed Galton most strikingly. To meet the objection of environmentalists Wood investigated the royal families of Europe, where environment is uniformly favourable; and if opportunity rather than hereditary capacity were all that is necessary for success, most members of this class should have been successful. Wood's studies led to a contrary conclusion. Royal genius and ability is a definite "family matter," concentrated in isolated chains of closely-related individuals. One chain centres in Frederick the Great, another in Isabella of Spain, a third in William the Silent, and a fourth in Gustavus Adolphus. Moreover, inferiority in royalty is equally segregated, royal dullards and degenerates also running in families.

If one accepts heredity as the all-important factor in the production of a superior or inferior people, and if one is disposed to regard the failure of civilizations in the past as due to a gradual deterioration of their races, what stage has been reached by western civilization ?

Many thoughtful people consider that our civilization has already passed its zenith, and that the rapid parabolic descent has already begun. We have heard ourselves described as a C3 nation, in that the standard of later recruits was extremely low both mentally and physically in the War ; and even before the War between 300 and 400 men in every 1,000 were rejected as unfit for military service. An investigation was made in Sheffield in 1919 as to the mental efficiency of its manual workers, "men and women of Yorkshire and Lancashire, which have a reputation for the vigour and achievement of their sons and daughters. 866 men and women, in equal numbers, were judged by their personalities and life histories as to their ability to cope with the problems of life in a satisfactory manner." Of these, 22 per cent. were found to be of character, often of fine character, and quite able to deal with life and its problems ; 70 per cent. were without positive qualities and unfit to vote ; 8 per cent. were a bad lot—vicious and depraved.

The statistical investigations by Majors Yoakam and Yerkes in America of 1,700,000 males examined for war service by the Binet-Simon tests is but one of many evidences that all is not well with western civilization from the point of view of either physical or mental capacity. If these 1,700,000 are assumed to be average specimens of a population of a hundred million, then the average mental age of Americans is only about that of a child of fourteen, forty-five million will never develop beyond the mental capacity represented by the intelligence of a child of twelve years, only one-eighth of the population will ever show superior intelligence, and less than one-twentieth can be considered talented.

Compare this, as Stoddart says, with the population of England, one twenty-fifth of this size, which produced the men of the Elizabethan age : Shakespeare, Ben Jonson, Marlowe, Harvey, Bacon, Drake, Raleigh and Hawkins, and these are but the individual geniuses. Besides them there grew up a splendid school of dramatists, a fine architectural style, great pioneers in science and philosophy, brilliant navigators and explorers. In comparison with this record the geniuses that arose in the World War would be hard to find.

To quote from Popenoe and Johnson in *Applied Eugenics :* "Men differ — these differences are inherited, so that the make-up of a race can be changed by any method which will alter the relative proportion of the contributions which different classes of men make to the following generation. From a eugenist point of view it is enough to know that mental and physical differences are inherited—how much is not important here. Research has advanced far beyond the primitive Mendelism conceived fifteen years ago. The modern view being that, instead of allotting one factor to each characteristic, students now believe that each individual characteristic of the organism is induced by the action of an indefinitely large number of factors, and are now forced to the belief that each factor affects an indefinitely large number of characters. Thus one factor in the sweet pea changes not only height but colour of foliage, length of internodes, size and arrangement of flowers, time of flower opening, fertility and variability. Feeble-mindedness is not due to a unit character. Intelligence is due to the inheritance of a vast but indeterminate number of factors of various kinds. Hence all grades from the genius to the idiot ; but much psychological analysis must be done before the inheritance of feeble-mindedness is solved. However, it can be said of mental traits as others, that 'like tends to produce like'—low-grade mentality generally comes from ancestry of low mentality and vice versa. 'A good tree cannot bring forth evil fruit, neither can a corrupt tree bring forth good fruit.' "

In the past natural selection has been responsible for the improvement of the race. Popenoe and Johnson say that "during the last century the philanthropic spirit and the progress of medicine have done a great deal to interfere with natural selection. In some ways selection of the human race has almost ceased, in many ways it is actually reversed ; that is, it results in the survival of the inferior rather than the superior.

In the olden days the criminal was summarily executed ; the weakly child died soon after birth through lack of proper care and medical attention ; the insane were dealt with so violently that if they were not killed by the treatment they were at least left hopelessly incurable, and had little chance of becoming parents. Harsh measures all of these, but they kept the germ plasm of the race reasonably purified.

To-day the inefficient, the wastrel, the physical, mental and moral cripples are carefully preserved at public expense. The criminal is turned out on parole after a few years to become the father of a family.

The insane are discharged as "cured," again to take up the duties of citizenship. The feeble-minded child is painfully "educated," often at the expense of his normal brother and sister. In short, the undesirables of the race, with whom the bloody hand of natural selection would have made short work of early in life, are now nursed along to old age. "Of course, one would not have it otherwise with respect to the prolongation of life. To expose deformed children, as the Spartans did, would outrage our moral instincts; to chloroform the incurable is a proposition that almost everyone condemns; but the philanthropic spirit is leaving a staggering bill to be paid for by posterity."

Nowadays, while failing to reproduce themselves, the function of the superior people is to support the inferior, who reproduce as three to one of the superior (Loudon). The scientific man has on the average about seven-tenths of an adult son. Davenport, the biologist, calculated that at the present rate of reproduction a thousand Harvard graduates of to-day would have only fifty descendants two centuries hence, whereas a thousand Roumanians to-day in Boston, at their present rate of breeding, would have 100,000 descendants in the same space of time. And about the same holds good for the graduates of our older Universities and the proletariat.

Plato in his *Republic* said of Æsculapius that "he revealed the healing art for the benefit of those whose constitutions were naturally sound, but that where the constitution was thoroughly diseased to the core he did not attempt to prolong a miserable existence . . . and suffer his patients to beget children, in all probability, as diseased as themselves."

There is in existence to-day, and there has been for years, a perverted humanitarianism. It is that which would support the wastrel at public expense; that insists on the preservation at birth of the Mongolian idiot; that would multiply the insane and mental deficient at the expense of the efficient, rather than sterilize them to reproduce no more. It cries aloud at vivisection, preferring that the flesh and blood of others should continue to suffer rather than put an animal through an experiment — no more painful than man is constantly being submitted to by surgery. This humanitarianism would abolish corporal and capital punishment. It is often like that of an old lady I once knew who was so humane that she could not even kill a flea, but kept for them a special old red flannel petticoat. It is that mistaken philanthropy which supplies the funds even for the provision of pocket-handkerchiefs for the heathen. Surely this type of humanitarianism is anti-social and anti-racial.

The question I would ask you to consider is: Has medicine interfered with natural selection? I think one can definitely say that medicine, scientific or otherwise, has concerned itself, primarily and always, with the preservation of the individual, irrespective of his or her value to the race. The race has not been even a secondary consideration in its activities. It has striven to make and been often lauded for its discoveries for the preservation of the individual, whether they be fit or unfit, desirable or undesirable. Some authorities are of the opinion that the Great Plague was chiefly a disease of the "masses," the less intelligent people being destroyed in enormous numbers, probably with benefit to the stock. Small-pox was one of these eliminators, and the question as to which is the better eugenist, the public health medical service with its vaccine or the anti-vaccinationist, gives one pause. The anti-vaccinationist exists mainly among the least intelligent of the people. It has been stated that fifty per cent. of these are unvaccinated. Given a thoroughly virulent small-pox epidemic, is it not probable that the anti-vaccinationist might make an enormous contribution, a holocaust, to racial betterment, proving himself, unintentionally, a better eugenist than medicine with its vaccine ?

The achievements of the public health authorities in reducing the infant death-rate cannot be regarded with complete equanimity, since they chiefly affect the worst stratum of society, who already out-reproduce the best as three to one. The child welfare clinic is not without its drawbacks. Nature removes many weaklings in the first year, but numbers of them are now triumphantly preserved to reproduce their like.

Is the fight against tubercle so particularly desirable ? The mortality of the white man's plague has, in some places, fallen fifty per cent. In other words, this fifty per cent. may now survive for a longer age of reproduction ; the diathesis is thereby spread over a greater and still more great number of the population. Is this an advantage to the race ?

The result of many of the activities of the public health medical service appears only too often to be a reversal of natural selection. It might conceivably be better for the population if all those with hereditary diseases died before the reproductive age, or were rendered sterile. There seems no sound reason, for instance, that justifies the survival of the congenitally hydrocephalic, the Mongolian idiot, the achondroplasic, often a misery to their parents and to themselves. Is it not a perverted humanitarianism, an actual cruelty to the individual thus afflicted and to the normal members of the family ? The discovery of insulin may prove detrimental to the race, by increasing the number of diabetics to the extent of the inheritability of the disease : *already* many are now surviving to reproduce. If heredity holds in cretinism, our knowledge of the value of thyroid medication may not benefit human stock.

The greatest canker in our modern civilization are the mental deficients, who with the insane are slowly and steadily levelling down the general national capacities. Three hundred thousand of them in England, at a low estimate, in and through the general population rapidly multiplying their like, and, which is even more serious, producing the sub-man as ten to one of themselves. Has medicine made any serious effort to grasp and root out this evil ?

Nature has given us natural selection to maintain a high standard both physical and mental. Can one honestly say that medicine has aided and abetted her ? Medicine appears, on the contrary, to be thwarting Nature in many ways by encouraging the survival of the unfit, for which a retribution is already established in the preponderance and swamping of the good by evil stock. What can one hope for western civilization with the scientific outlook such as it is ?

Science as a whole, and scientific medicine in particular, is out of control, it moves regardless of the race, and not infrequently wields its knowledge like a boy with a loaded gun. It is time that medicine regarded its duty to the race as its chief and highest function, for a raising of the national mental and physical standards means greater health and happiness for all. Can we say that medicine is not responsible for the existence of a vast number of our sick ? If this is so, is not medicine therefore responsible for the expenditure of housing and treating them. It is the duty of the medical profession to set its house in order, and by its constant efforts to improve the race to be able to show as a criterion of its work that the need for hospital beds grows less and less ; to demand more and more is but a measure of its incompetence. It would be indeed an achievement to diminish the necessity for beds by half and to eliminate the ament.

The problem to be solved is the raising and maintaining of the people at a high level of mental and physical capacity, on which civilization depends, and compared with which all other questions whatsoever are of little import. A satisfactory solution to this is vital to the arrest of the parabola's descent. It is the only means by which man, by his own reason and effort, can ascend to the heights, for without doubt there is a plasticity within him which may lead to heights undreamt of.

The education of the public on the vital importance of race maintenance and improvement is a paramount need, to which the medical profession as a whole should devote some of its energies. It should at the same time concentrate on practising that which it should preach, and seriously endeavour to eliminate its own means of existence, by attempting to produce an A1 rather than to encourage a C3 nation.

Plato says in his *Republic :* " Where can you find a more signal proof that a low and vicious education prevails in a State than in the fact that first-rate physicians are in request . . . even among those who lay claim to the birth and breeding of gentlemen." The aim of the profession should be to produce such a people that the demand for sick accommodation is reduced to a minimum.

There are two means by which this can be approached. First and most easily achieved, once public interest is aroused, the prevention of procreation by the grossly unfit mentally, the feeble-minded, who are, as Dr. Fisher says, responsible for the whole burden of disease, defect and pauperism. Secondly, by providing each and every means that will discourage the reproduction of the sub-man, and, the corollary to this, the encouragement of the birth of those of superior intelligence. Both these are duties of the State in co-operation with medicine.

The ament must be dealt with first by one of two methods, namely segregation and sterilization. Complete segregation of aments can be at once set aside as impracticable, from the point of view of expense and uneducated public opinion. But partial segregation—which already exists for the insane, the mental defectives, the criminal and the potential criminal—should be complementary and supplementary to sterilization.

Compulsory sterilization is at present out of the question for the feeble-minded and insane, but voluntary sterilization *already* exists. In California the parents of defective children are beginning to bring these for sterilization to prevent "accidents," and every encouragement should be given by medicine and the State to this, as a means of public education and racial improvement. In the Swiss Canton de Vaud in October, 1928, was passed a law that people suffering from mental infirmity, who can only produce tainted children, shall be dealt with by a "Council of Health" by such means as shall prevent their propagating. It is probable, says Fisher, that adequate measures to prevent reproduction by aments would reduce them by one-third in the first generation.

The marriage of mentally-diseased people should be forbidden as long as they can reproduce, and the discharge from an institution of the potentially fertile should be forbidden, save after sterilization. The same should hold for the deaf-mute, the congenitally blind, those with pronounced tubercle, and others suffering from hereditary diseases. Popenoe says that sterilization will prevent the birth of few, if any, superior people, but of many inferior. The sterilization of mental deficients runs no risk of losing genius or great talent. A beginning must be made on the ament by sterilization, and though it does not touch the great mass of sub-men already existing, who have arisen from the feeble-minded, if the ament be their breeding-ground it will at least lessen its fertility.

It has, perhaps, ill behoved me to venture on so vast a subject such as this, to put before you a problem that you all must know so well, and many of you be far better able to pass judgment on than myself ; which, moreover, has been voiced by far more able members of our beloved profession. My excuse must be that the subject has long interested me, and I felt moved to present to you, however redundantly and inadequately, a crude and superficial presentation of what none can deny is the most important question of all time.

One has a vision of a world in which science has lost its greatest opportunity, in which civilization has again failed, order degenerating into chaos, such as now exists in Russia, to be followed by barbarism and even savagery. But a vision also one has of a world from which men, such as myself, have been long since eliminated, where the eminent of to-day are as the sub-man, and where men with their great foster-mother Medicine walk as gods.

